# Molecular epidemiology study of programmed death ligand 1 and ligand 2 protein expression assessed by immunohistochemistry in extensive-stage small-cell lung cancer

**DOI:** 10.3389/fonc.2023.1225820

**Published:** 2024-01-09

**Authors:** Torben Steiniche, Jeanette Baehr Georgsen, Peter Meldgaard, Anne C. Deitz, Mark Ayers, M. Catherine Pietanza, Ke Zu

**Affiliations:** ^1^ Department of Pathology, Aarhus University Hospital, Aarhus, Denmark; ^2^ Department of Oncology, Aarhus University Hospital, Aarhus, Denmark; ^3^ Merck & Co., Inc., Rahway, NJ, United States

**Keywords:** extensive-stage small-cell lung cancer, PD-L1, PD-L2, biomarker, prevalence

## Abstract

**Objectives:**

Prevalence of tumor PD-L1 expression in extensive-stage small-cell lung cancer (ES-SCLC) is variable, and data on PD-L2 expression are limited. The prognostic values of these biomarkers are not well understood. The current study was conducted to address these data gaps.

**Methods:**

A retrospective cohort study of Danish patients with histologically confirmed ES-SCLC and evaluable tumor samples who were receiving usual care before the introduction of immunotherapy was conducted. Protein expression of PD-L1 and PD-L2 was determined by immunohistochemistry (IHC) using the PD-L1 IHC 22C3 pharmDx assay and a PD-L2 IHC assay using a propriety mouse monoclonal antibody. A combined positive score (CPS) of ≥1 was used to define biomarker positivity. Kaplan-Meier plots and Cox proportional hazard models were employed to assess the relationship between PD-L1 and PD-L2 protein expression and OS.

**Results:**

Among 80 patients, 31% (n=25) and 36% (n=29) had disease positive for PD-L1 and PD-L2, respectively. Overall, 85% (n=68) of patients had concordant PD-L1/PD-L2 status; 26% (n=21) had double positive disease (both PD-L1 and PD-L2 CPS ≥1) and 59% (n=47) had double negative disease (both PD-L1 and PD-L2 CPS <1). PD-L1 and PD-L2 positivity were each associated with longer OS (unadjusted hazard ratios [HRs], 0.35 [95% CI, 0.21−0.61] and 0.50 [95% CI, 0.31−0.82]); the associations persisted after adjustment for several known prognostic factors (HRs, 0.41 [95% CI, 0.22–0.75] and 0.44 [95% CI, 0.25–0.79] for PD-L1 and PD-L2 positivity, respectively). When evaluating OS in patients with double positive disease, unadjusted and adjusted HRs for double positive compared with double negative were similar to those with only PD-L1 or PD-L2 positivity (unadjusted HR, 0.36 [95% CI, 0.20–0.64]; adjusted HR, 0.36 [0.18−0.73]).

**Conclusion:**

PD-L1 and PD-L2 positivity were observed in approximately one-third of assessed ES-SCLC tumor samples and were highly congruent. Patients with PD-L1 and PD-L2 positivity, alone or combined, were associated with longer OS, independent of other prognostic factors.

## 1 Introduction

Small-cell lung cancer (SCLC) is a neuroendocrine tumor typically associated with smoking that accounts for approximately 15% of all lung cancers (1–3). The majority of patients with SCLC present with extensive-stage (ES) disease at initial diagnosis (4). Until recently, the standard of care for previously untreated extensive-stage small-cell lung cancer (ES-SCLC) was etoposide-platinum chemotherapy (5). Although ES-SCLC is often initially sensitive to chemotherapy, most patients experience disease recurrence or progression after primary treatment, with reported median overall survival (OS) of approximately 10 months and 5-year survival rate of approximately 2% (3, 6).

Programmed cell death protein 1 (PD-1) is an immune checkpoint protein expressed on T cells and other immune cells. The binding of one of its ligands, programmed death ligand 1 (PD-L1) or programmed death ligand 2 (PD-L2), inhibits T-cell activation, cytokine production, and cytotoxic activity (7). The receptor-ligand interaction between PD-1 and PD-L1 and PD-L2 is a major pathway used by tumors to suppress immune control and is consequently a therapeutic target for immunotherapy (8–10).

Results from phase 3 studies have demonstrated improvement in outcomes with addition of anti−PD-1 or anti−PD-L1 agents to platinum-based chemotherapy in patients with previously untreated ES-SCLC. In the IMpower133 study, the combination of atezolizumab, an inhibitor of PD-L1, with etoposide and carboplatin significantly improved OS and progression-free survival (PFS) in patients with ES-SCLC compared to placebo with etoposide and carboplatin (9). Similarly, in the CASPIAN study, durvalumab, also a PD-L1 inhibitor, in combination with etoposide and cisplatin or carboplatin significantly improved OS in comparison to etoposide with cisplatin or carboplatin in patients with previously untreated ES-SCLC (10). In the KEYNOTE-604 study, pembrolizumab, a PD-1 inhibitor, combined with etoposide plus carboplatin or cisplatin significantly improved PFS and demonstrated a trend in improved OS compared with etoposide plus carboplatin or cisplatin in patients with previously untreated ES-SCLC (11). Based on data from the IMpower133 and CASPIAN trials, the combinations of atezolizumab and durvalumab with etoposide-platinum chemotherapy are approved by regulatory agencies globally and have been included in the National Comprehensive Cancer Network management guidelines version 2.2023 for the treatment of patients with previously untreated ES-SCLC (5, 12). Notably, the combinations of atezolizumab and durvalumab with etoposide-platinum chemotherapy have been the first to change the treatment paradigm in SCLC in decades (12).

The prevalence of PD-L1 expression in SCLC reported in the literature varies widely. Previous studies evaluating the presence of PD-L1 in primary and metastatic tumor tissue and tumor-infiltrating lymphocytes using IHC or RNA expression have reported expression ranging from 0% to 75.0% (13–17). PD-L1 expression has been reported in 12.6% to 62.3% of patients with ES-SCLC (11, 15, 18, 19). Notably, these data from patients with ES-SCLC are derived from a relatively small number of tumor samples. The observed variations are likely due to differences in the applied IHC assays and the lack of standardization for PD-L1 assessment between different studies, definition of positivity thresholds, and scoring platforms. Evidence for the prognostic value of PD-L1 expression in SCLC is equivocal (14, 19–22). A meta-analysis of 9 studies and an analysis of retrospectively collected SCLC tissue samples assessed by multiplexed quantitative immunofluorescence found that PD-L1 expression was not a significant predictor of poor OS (14, 22). However, other studies have shown associations between PD-L1 expression and clinical outcomes. A retrospective analysis of SCLC patient samples found that those with PD-L1–positive tumors had significantly longer OS than those with negative tumors, while another retrospective study found that PD-L1 expression was associated with significantly poorer PFS and OS in an SCLC cohort (20, 21). Furthermore, a separate retrospective study found that patients with PD-L1–positive tumors had longer OS than the PD-L1–negative group, although this finding was only significant when assessed by the PD-L1 SP142 assay and not when assessed using the PD-L1 SP263 assay or the PD-L1 IHC 22C3 assay (19).

In contrast to PD-L1, data on PD-L2 expression and its prognostic value in SCLC are sparse, and nonexistent in ES-SCLC specifically. A retrospective analysis of 38 patients with surgically resected SCLC reported that 37% of tumor samples were positive for PD-L2 at a cutoff of 1% of all carcinoma cells as assessed by IHC (23). No significant associations were observed between PD-L2 positivity and specific clinicopathologic characteristics or between PD-L2 positivity and disease-free survival and OS (23).

As anti–PD-(L)1 therapies have demonstrated efficacy in patients with PD-L1−negative tumors in certain cancer types (24), it has been hypothesized that inhibition of the interaction between PD-1 and PD-L2 may be involved in mediating response in these patients (25). However, the prognostic value of PD-L1 and PD-L2 or the coexpression of these biomarkers is not well understood in patients with ES-SCLC. We conducted an observational, retrospective cohort study to evaluate the prevalence of PD-L1 and PD-L2 protein expression or coexpression in tumor tissue specimens from Danish patients with ES-SCLC. An exploratory objective was to determine the prognostic value of PD-L1 and PD-L2 protein expression or coexpression for patients with ES-SCLC receiving usual care in Denmark before the introduction of immunotherapy.

## 2 Materials and methods

### 2.1 Study design and patients

This observational, retrospective study was conducted among patients with ES-SCLC receiving usual care in the clinical setting in Denmark. Patients were identified through the Aarhus University pathology database and had a recorded diagnosis of ES-SCLC between January 1, 2000, and December 31, 2015. Due to limited tumor tissue availability from eligible patients with ES-SCLC in the database, some patients with ES-SCLC from a previous molecular epidemiology study of rare tumors conducted at the same site were also included in this analysis (26).

Eligible patients were at least 18 years of age at the time of diagnosis and had a histologically confirmed diagnosis of SCLC, with a tumor tissue sample at ES disease (T any, N any, M1a/b based on the American Joint Committee on Cancer manual, 7th edition) of sufficient quality and quantity for PD-L1 and PD-L2 IHC testing. Patients were excluded if they had a history of prior malignancy (except basal cell carcinoma of the skin, superficial bladder cancer, squamous cell carcinoma of the skin, or *in situ* cervical cancer or had undergone potentially curative therapy with no evidence of disease recurrence for 5 years); diagnosis of other primary tumor at the time of SCLC diagnosis; or if their tumor samples were derived from bone metastases, previously frozen tumor samples, or cell blocks that had not been validated with the immunohistochemistry assays used in this study. Because this was an observational epidemiologic study using secondary data with no active recruitment of patients and no drug administration, investigators received a waiver of patient consent from the regional Ethics Committee (Videnskabsetiske Komitéer for Region Midtjylland, IRB # 1-16-02-206-19).

### 2.2 PD-L1 and PD-L2 assessment

**Figure 1:**
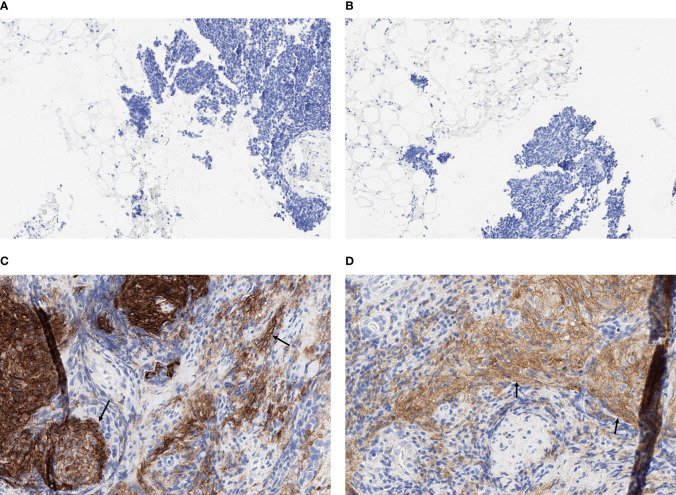
Representative fields (x20) of IHC staining for PD-L1 and PD-L2 in SCLC tumor tissue samples. **(A)** PD-L1 negative (CPS = 0), **(B)** PD-L2 negative (CPS = 0), **(C)** PD-L1 positive (CPS = 40), and **(D)** PD-L2 positive (CPS = 40). Arrows indicate representative areas of positive staining for PD-L1 **(C)** and PD-L2 **(D)**.

Formalin-fixed paraffin-embedded (FFPE) tumor blocks from core or excisional biopsies or resected tumor tissue were retrieved for eligible patients. Four unstained slides (2 for PD-L1 testing and 2 for PD-L2 testing) and 1 matched hematoxylin and eosin−stained slide (shared across the assays), each with >100 tumor cells, were the minimum required. Tissue slides, each 4 µm thick, were cut and processed by the investigators at the Aarhus University Hospital in Denmark according to commercial laboratory specifications for IHC analyses. Expression of PD-L1 and PD-L2 was assessed at NeoGenomics Laboratories Inc. (Fort Myers, FL, USA) using the PD-L1 IHC 22C3 pharmDx assay (Agilent Technologies, Carpinteria, CA, USA) and PD-L2 IHC assay using a propriety mouse monoclonal antibody (clone MEB123.3G2.038), respectively. Expression was quantified using a combined positive score (CPS), which was calculated as the number of cells (tumor cells, lymphocytes, and macrophages) stained positive for PD-L1 or PD-L2 divided by the total number of viable tumor cells, multiplied by 100. Representative IHC staining of PD-L1 and PD-L2 in SCLC tumor tissue samples are shown in **Figures 1**; **S1**. Cutoffs of CPS ≥1, ≥10, and ≥20 were explored for defining biomarker positivity.

### 2.3 Study variables

Information on patient demographic and clinical characteristics, treatment, and clinical outcomes, was collected from the Aarhus pathology database and patient medical records as available, merged with dichotomized biomarker data for PD-L1 and PD-L2, and fully anonymized before analysis. Key variables included date and age of initial SCLC diagnosis, date and age of ES-SCLC diagnosis, smoking history, Eastern Cooperative Oncology Group performance status (ECOG PS) score, SCLC stage at initial diagnosis, date of SCLC specimen collection, stage of SCLC at time of specimen collection, treatment chemotherapy prior to specimen collection, site of metastases (liver or brain), chemotherapy regimen, response to first-line chemotherapy, previous radiation therapy, and lactate dehydrogenase (LDH) level.

### 2.4 Statistical analysis

Descriptive analyses were conducted for the prevalence of PD-L1 and PD-L2 protein expression or coexpression and summarized using counts and proportions. Sensitivity analyses were performed with restriction of the study population to patients whose tumor specimens were collected at ES (i.e., including limited-stage [LS]-SCLC progression to ES-SCLC or ES-SCLC at initial diagnosis).

Follow-up time was defined as the time from the initial diagnosis of SCLC to the date of death (due to any cause) or the date of the last recorded visit if the patient was alive at the time of data extraction. The exploratory outcome of interest was overall survival (OS), defined as the time from SCLC diagnosis to death due to any cause. Patients without documented death at the time of the last follow-up were censored. Kaplan-Meier plots with log-rank tests were used to examine the relationship between median OS and biomarker status. Cox proportional hazards models were employed to explore associations between PD-L1 and PD-L2 expression or coexpression and OS, with adjustment for important covariates as available (e.g., age, gender, smoking, performance score, LDH). Covariates included in the multivariate models were determined based on a stepwise variable selection process: covariates with a *P* value < 0.2 from the univariate analysis were included in the stepwise variable selection and removed when their *P* values were > 0.1 in the multivariate models. The variables of age at SCLC diagnosis (continuous) and ECOG PS were forced into the multivariate models during the stepwise variable selection process. Additional sensitivity analyses were conducted in which OS was defined as the time from ES-SCLC diagnosis to death due to any cause.

## 3 Results

### 3.1 Patients

A total of 80 patients with a diagnosis of ES-SCLC between January 1, 2000, and December 31, 2015 were included in the study. Tissue was obtained for 70 out of 80 patients at the time of diagnosis with ES-SCLC; 10 patients’ specimens were collected when they were initially diagnosed with limited stage disease.

Baseline demographic and clinicopathologic characteristics are summarized in **Table 1**. Among the 80 patients with ES-SCLC, the mean (SD) age at ES-SCLC diagnosis was 65.5 (9.92) years, 50% (n=40) were male, and the majority (71%; n=57) had previously or were currently receiving chemotherapy. The median follow-up time for the study population is 10.6 months.

**Table 1:** Demographic and clinicopathologic characteristics of patients with ES-SCLC in Denmark.

	All Patients With ES-SCLC^a,b^ N = 80	Patients With ES-SCLC Specimens^a,c^ N = 70
Age at SCLC diagnosis, mean (SD), y	65.2 (9.91)	65.9 (9.71)
Age at ES-SCLC diagnosis, mean (SD), y	65.5 (9.92)	66.1 (9.75)
Male	40 (50)	35 (50)
Follow-up time, median (range), m	10.6 (0.2–71.3)	8.2 (0.2–71.3)
Tissue collection date		
2000−2009	23 (29)	20 (29)
2010−2014	57 (71)	50 (71)
Smoking history		
Current	38 (48)	35 (50)
Former	18 (23)	14 (20)
Unknown	24 (30)	21 (30)
ECOG PS		
0	10 (13)	10 (14)
1	22 (28)	17 (24)
≥2	27 (34)	25 (36)
Unknown	21 (26)	18 (26)
Stage of SCLC at diagnosis		
Limited (I, II, III)	19 (24)	9 (13)
Extensive (IV)	61 (76)	61 (87)
Stage of SCLC at time of specimen collection		
LS-SCLC	10 (13)	NA
LS-SCLC with progression to ES	9 (11)	9 (13)
ES-SCLC at initial diagnosis	61 (76)	61 (87)
Metastasis		
Any metastases	76 (95)	66 (94)
Liver	18 (23)	15 (21)
Brain	21 (26)	18 (26)
Bone	21 (26)	17 (24)
Pleura	2 (3)	2 (3)
Peritoneum	2 (3)	2 (3)
Skin	3 (4)	2 (3)
Distant lymph node	27 (34)	26 (37)
Other	19 (24)	16 (23)
Any liver or brain metastases	37 (46)	32 (46)
Treatment prior to specimen collection		
Chemotherapy exposed	9 (11)	9 (13)
Chemotherapy naive	69 (86)	59 (84)
Unknown	2 (3)	2 (3)
Prior or current chemotherapy		
Yes	57 (71)	49 (70)
Etoposide-platinum− containing regimen	53 (93)	47 (96)
Irinotecan- containing regimen	0	0
No	10 (13)	8 (11)
Unknown	13 (16)	13 (19)
1L chemotherapy		
Response to 1L chemotherapy	57 (71)	49 (70)
Sensitive	40 (70)	35 (71)
Insensitive	7 (12)	5 (10)
Unknown	10 (18)	9 (18)
No response	20 (25)	18 (26)
Unknown	3 (4)	3 (4)
Previous radiation therapy		
Yes	5 (6)	5 (7)
No	62 (78)	52 (74)
Unknown	13 (16)	13 (19)
Lactate dehydrogenase		
Normal	18 (23)	15 (21)
Elevated	27 (34)	24 (34)
Unknown	35 (44)	31 (44)

1L, first line; ECOG PS, Eastern Cooperative Oncology Group performance score; ES, extensive stage; LS, limited stage; NA, not available; SCLC, small-cell lung cancer.

a All data are n (%) unless otherwise noted.

b 80 patients with a diagnosis of ES-SCLC.

c Tissue was obtained for 70 out of 80 patients at the time of diagnosis with ES-SCLC.

### 3.2 Prevalence of PD-L1 and PD-L2 expression

Among the total population included in the primary analysis, 31% (n=25) of tumors were PD-L1–positive at CPS ≥1, 4% (n=3) at CPS ≥10, and 1% (n=1) at CPS ≥20. For PD-L2 expression, 36% (n=29) of tumors were positive at CPS ≥1, 9% (n=7) at CPS ≥10, and 8% (n=6) at CPS ≥20 (**Table 2**). Because of the low proportion of patients with PD-L1 and PD-L2 at cutoffs of CPS ≥10 and ≥20, CPS ≥1 was selected to define positive expression of PD-L1 and PD-L2 specimens in subsequent analyses; CPS <1 defined PD-L1– and PD-L2–negative specimens.

**Table 2:** PD-L1 and PD-L2 protein expression and coexpression in tumor specimens from Danish patients with ES-SCLC.

Biomarker	All Patients With ES-SCLC^a^ N = 80	
PD-L1 expression, CPS		
≥1	25 (31)	
<1	55 (69)	
≥10	3 (4)	
<10	77 (96)	
≥20	1 (1)	
<20	79 (99)	
PD-L2 expression, CPS		
≥1	29 (36)	
<1	51 (64)	
≥10	7 (9)	
<10	73 (91)	
≥20	6 (8)	
<20	74 (93)	
PD-L1 and PD-L2 Protein Coexpression	PD-L1 CPS ≥1	PD-L1 CPS <1
PD-L2 CPS ≥1	21 (26)	8 (10)
PD-L2 CPS <1	4 (5)	47 (59)

CPS, combined performance score; ES-SCLC, extensive-stage small-cell lung cancer; PD-L1, programmed death ligand 1; PD-L2, programmed death ligand 2.

a All data are n (%).

Using the cutoff point of CPS ≥1, 26% (n=21) of the total number of patients with ES-SCLC (i.e., n=80) had tumors that were positive for both PD-L1 and PD-L2 (double positive) and 59% (n=47) were negative for both PD-L1 and PD-L2 (double negative). Five percent (n=4) of tumors were positive for PD-L1 only (PD-L1 single positive) whereas 10% (n=8) were positive for PD-L2 only (PD-L2 single positive; **Table 2**). For patients who were diagnosed with LS-SCLC and progressed to ES-SCLC (n=9), 5 (20%) had tumors that were PD-L1 positive.

### 3.3 OS and PD-L1 expression

There were few major differences in clinicopathologic characteristics when assessed by PD-L1 expression (CPS ≥1 vs <1). The characteristics in which differences by PD-L1 expression were observed included initial diagnosis of ES-SCLC, which was identified in 84% of patients with PD-L1–negative tumors and 60% with PD-L1–positive tumors, and elevated lactate dehydrogenase levels in 16% and 42%, respectively (**Table S1**). Of 25 patients whose tumors were positive for PD-L1, 22 had died, and of 55 patients whose tumors were negative for PD-L1, 54 had died. Median OS, defined as the time from initial SCLC diagnosis to death, was 19.4 months (95% CI, 13.3−28.0 months) in patients with PD-L1–positive tumors and 7.5 months (95% CI, 3.0−10.9 months) in patients with PD-L1–negative tumors. In patients with PD-L1–positive disease, estimated OS rates were 72.0% at 1 year, 22.0% at 3 years, and 11.0% at 5 years. In patients with PD-L1–negative disease, estimated OS rates were 32.7% at 1 year, 2.2% at 3 years; there were no surviving patients at 5 years (**Table 3**, **Figure S2**). The hazard ratio (HR) for OS among patients assessed as PD-L1–positive versus those assessed as PD-L1–negative was 0.35 (95% CI, 0.21−0.61) in unadjusted analyses. In the multivariate analysis, adjustment for age and stage at SCLC diagnosis, ECOG PS, treatment prior to specimen collection, LDH level, and smoking history did not impact the observed association between PD-L1 expression status and OS (HR, 0.41; 95% CI, 0.22−0.75).

**Table 3:** OS and PD-L1 protein expression in tumor specimens from Danish patients with ES-SCLC.

	PD-L1–negative disease	PD-L1–positive disease
N	55	25
No. of deaths	54	22
1 year survival rate, %	32.7	72.0
3 year survival rate, %	2.2	22.0
5 year survival rate, %	Not reached^a^	11.0
Median OS (95% CI), mo	7.5 (3.0–10.9)	19.4 (13.3–28.0)
Crude HR (95% CI)	Ref.	0.35 (0.21–0.61)
Adjusted HR^b^ (95% CI)	Ref.	0.41 (0.22–0.75)

a No patient remained alive after 5 years.

b Multivariate model adjusted for age at SCLC diagnosis, ECOG PS, stage of SCLC at initial diagnosis, specimen exposure to chemotherapy, LDH, and smoking history.

The HRs for OS for patients with PD-L1–positive disease versus PD-L1–negative disease presented above (where OS was defined as the time from initial SCLC diagnosis to death) were similar to those in the total population when OS was defined as the time from diagnosis of ES-SCLC to death in all patients with ES-SCLC, or among the 70 patients with ES-SCLC tumor specimens (data not shown).

### 3.4 OS and PD-L2 expression

Similar to PD-L1 expression, the majority of demographic and clinicopathologic characteristics did not differ by PD-L2 expression status, except that patients with disease positive for PD-L2 had lower LDH levels (**Table S2**). Of 29 patients with disease positive for PD-L2, 27 had died and of 51 patients negative for PD-L2, 49 had died. Median OS, defined as the time from initial SCLC diagnosis to death, was 17.2 months (95% CI, 9.3−22.6 months) in patients with PD-L2–positive disease and 7.5 months (95% CI, 2.3−11.3 months) in patients with PD-L2–negative disease. Estimated 1-, 3-, and 5-year OS rates were 62.1%, 15.5%, and 7.8%, respectively, in patients who were PD-L2–positive and 35.3%, 4.5%, and 2.2%, respectively, in patients who were PD-L2–negative (**Table 4**, **Figure S3**). The HR for OS for PD-L2–positive versus PD-L2–negative patients was 0.50 (95% CI, 0.31−0.82) in unadjusted analyses and 0.44 (95% CI, 0.25−0.79) in multivariate analysis adjusted for age and stage at SCLC diagnosis, ECOG PS, treatment prior to specimen collection, LDH level, and smoking history. The observed inverse association between PD-L2 expression and OS persisted in sensitivity analyses when OS was defined as the time from diagnosis of ES-SCLC to death or the population was restricted to patients with ES-SCLC tumor specimens.

**Table 4:** OS and PD-L2 protein expression in tumor specimens from Danish patients with ES-SCLC.

	PD-L2–negative disease	PD-L2–positive disease
N	51	29
No. of deaths	49	27
1 year survival rate, %	35.3	62.1
3 year survival rate, %	4.5	15.5
5 year survival rate, %	2.2	7.8
Median OS (95% CI), mo	7.5 (2.3–11.3)	17.2 (9.3–22.6)
Crude HR (95% CI)	Ref.	0.50 (0.31–0.82)
Adjusted HR^a^ (95% CI)	Ref.	0.44 (0.25–0.79)

a Multivariate model adjusted for age at SCLC diagnosis, ECOG PS, stage of SCLC at initial diagnosis, specimen exposure to chemotherapy, LDH, and smoking history.

### 3.5 OS and PD-L1/PD-L2 coexpression

Most demographic and clinicopathologic characteristics did not differ among the 68 patients with PD-L1/PD-L2 coexpression status, with the exception of LDH level; a lower proportion of patients who had disease positive for both PD-L1 and PD-L2 had elevated LDH levels.

Of 21 patients with double positive disease, 19 had died, and of 47 patients who had double negative disease, 46 had died. Median OS, defined as the time from initial SCLC diagnosis to death, was 19.1 months (95% CI, 10.5−28.0 months) in patients who had double positive disease and 7.3 months (95% CI, 2.3−10.7 months) in those who had double negative disease. Estimated OS rates for patients with double positive disease were 71.4% at 1 year, 21.8% at 3 years, and 10.9% at 5 years and 31.9%, 2.7%, and not reached (i.e., no surviving patients), respectively, for patients with double negative disease (**Table 5**, **Figure S4**). The HR for OS was 0.36 (95% CI, 0.20−0.64) in unadjusted analyses and 0.36 (95% CI, 0.18−0.73) in multivariate analyses adjusted for age and stage of SCLC diagnosis, ECOG PS, liver or brain metastasis, treatment prior to specimen collection, LDH level, and smoking history. The HRs were not sensitive to the alternative definition of OS (ie, defined as the time from diagnosis of ES-SCLC to death) or to the restricted patient population with ES-SCLC tumor specimens only.

**Table 5:** OS and PD-L1 and PD-L2 coexpression in tumor specimens from Danish patients with ES-SCLC.

	PD-L1 and PD-L2 double negative disease	PD-L1 and PD-L2 double positive disease
N	47	21
No. of deaths	46	19
1 year survival rate, %	31.9	71.4
3 year survival rate, %	2.7	21.8
5 year survival rate, %	Not reached	10.9
Median OS (95% CI), mo	7.3 (2.3–10.7)	19.1 (10.5–28.0)
Crude HR (95% CI)	Ref.	0.36 (0.20–0.64)
Adjusted HR^a^ (95% CI)	Ref.	0.36 (0.18−0.73)

a Multivariate model adjusted for age at SCLC diagnosis, ECOG PS, stage of SCLC at initial diagnosis, liver or brain metastasis, specimen exposure to chemotherapy, LDH, and smoking history.

## 4 Discussion

This observational, retrospective study demonstrated the prevalence and prognostic value of PD-L1 and PD-L2 protein expression and their coexpression in ES-SCLC tumor specimens collected from Danish patients receiving standard of care before the introduction of immunotherapy. Overall, approximately 31% of patients had a tumor specimen with PD-L1 positivity (defined by a PD-L1 CPS ≥1), and 36% of patients had a tumor specimen with PD-L2 positivity (defined by a PD-L2 CPS ≥1). PD-L1 and PD-L2 coexpression were highly congruent in ES-SCLC, with more than 85% of patients with tumors that were either double positive (both PD-L1 and PD-L2 CPS ≥1) or double negative (both PD-L1 and PD-L2 CPS <1). There were no substantial differences in demographic or clinicopathologic characteristics by PD-L1 or PD-L2 expression or PD-L1/PD-L2 coexpression, with the exception of stage at SCLC diagnosis (for PD-L1 expression only) and LDH level. Patients with tumors positive for PD-L1 and PD-L2, assessed individually and by coexpression, had longer OS than those with PD-L1 and PD-L2 negative tumors, assessed individually and by coexpression; this was maintained after adjustment for demographic and clinicopathologic factors and in sensitivity analyses.

The prevalence of PD-L1 expression reported in the current analysis was broadly consistent with the range of values previously reported in patients with SCLC tumors (15, 16, 21). Of note, prior studies used different types of specimens (i.e., resected specimens, biopsies, primary and metastatic lesions), antibodies for IHC, and different assays to assess PD-L1 (e.g., BenchMark XT [Ventana Automated Systems, Inc., Tucson, AZ], PD-L1 immunohistochemical (SP263) assay on a Ventana BenchMark ULTRA automated staining platform, PD-L1 IHC 22C3 pharmDx assay [Agilent Technologies, Carpinteria, CA], and RNA sequencing analysis), which may in turn lead to variance in PD-L1 expression reported between different studies (11, 15, 21, 24). Compared with PD-L1, there are limited data evaluating the prevalence of PD-L2 expression in SCLC and no data for PD-L2 expression in ES-SCLC. Similar to our results, a prior retrospective study reported a prevalence of 37% of patients with PD-L2 positivity (defined by a cutoff value of 1%) in patients with SCLC (23). Notably, the prevalence of PD-L2 positivity reported in the aforementioned study was similar to the prevalence of PD-L2 positivity at CPS ≥1 observed in this study (36%). The high congruence between PD-L1 and PD-L2 may provide further insight into the mechanism of action of anti−PD-(L)1 therapies.

PD-L1 expression has been found to vary between tumor types. In a separate analysis of patients with 1 of 10 prespecified advanced rare tumor types previously conducted through the Aarhus University pathology network, the prevalence of PD-L1 positivity varied from 13% in patients with neuroendocrine tumors (n=30) to 86% in patients with vulvar carcinoma (n=44) (26). Variation in prevalence of PD-L1 expression has also been reported in studies evaluating other tumor types, including NSCLC (25), gastric cancer (27–29), urothelial cancer (30), triple-negative breast cancer (31), and cervical carcinoma (32). Additionally, the relationship between PD-L1 positivity and clinical outcomes has also been found to vary between tumor types (29, 31–36). This may be due to a varying role of PD-L1 as a prognostic factor (i.e., independent of treatment type), suggests the potential for differences between tumor types with respect to PD-L1–independent immune escape pathways, or may reflect differences in tumor heterogeneity, sample size, clinical stage, or the timepoint of PD-L1 measurement (37). As previously noted, various methodologies have been used in the assessment of PD-L1 expression and association with clinical outcomes in different tumor types in previous studies. Furthermore, differences in the number and type of previous therapies received may have confounded the assessment of the prognostic value of PD-L1 expression. As noted above, PD-L2 expression has also been reported to vary between different tumor types (38). The association of PD-L2 expression with clinical outcomes has also been found to vary between tumor types; in head and neck squamous cell carcinoma tumor specimens, high expression of PD-L2 was associated with shorter OS (39), whereas high expression of PD-L2 (at either 5% and 20% cutoffs) was found to prolong survival in patients with melanoma (40). PD-L2 expression was not associated with OS in patients with gastric cancer; patients with PD-L1/PD-L2 coexpression had better OS, although this did not reach statistical significance (41).

The majority of demographic and clinicopathologic characteristics did not differ by PD-L1 expression, PD-L2 expression, or coexpression; the exceptions to this included the stage of SCLC at diagnosis by PD-L1 expression only, and LDH level by PD-L1 expression, PD-L2 expression, and PD-L1/PD-L2 coexpression. The finding that more patients with PD-L1 positive tumors had normal LDH levels than those negative for PD-L1 is consistent with previous studies among patients with SCLC (21). The results from the current analysis indicate that PD-L1 and PD-L2 may have prognostic value in patients with ES-SCLC and suggest that this is likely not confounded by the demographic or clinicopathologic characteristics assessed in the current analysis.

In the current study, findings from exploratory analyses demonstrated that Danish patients with PD-L1–positive ES-SCLC receiving standard of care had longer OS than patients with PD-L1–negative tumors. There are limited data evaluating the prognostic value of PD-L1 expression in patients with ES-SCLC who received standard of care. With regards to PD-L2, in contrast to the current study, Takamori et al. (23) examined 38 patients who underwent surgical resection in Japan and found no difference in disease-free survival or OS between patients with PD-L2−positive and PD-L2−negative tumors, suggesting that PD-L2 does not provide additional prognostic value independent of PD-L1. However, with only 38 patients, Takamori et al. included less than half of the population in the current study (23). The smaller sample size, surgical resection of SCLC, and different antibody for PD-L2 analysis may have contributed to the lack of association observed between PD-L2 expression and survival.

The current study had several limitations. Firstly, this analysis was conducted at a single center and only included patients with ES-SCLC who received local (Denmark) standard of care, which may limit the generalizability of the results to other SCLC populations. Although the sample size was relatively small and not specifically powered for assessing the prognostic significance of the selected biomarkers, we observed substantive associations between OS and PD-L1 expression and PD-L2 expression that persisted after adjustment for multiple prognostic factors and in sensitivity analyses. As previously noted, the SCLC tumor specimens were collected between 2000 and 2014. Although IHC staining intensity can decrease over time during storage of tumor tissues (42), the age of the tumor specimen should not confound results in this analysis since the samples were fixed tissue blocks with slides freshly cut before IHC testing. Furthermore, the treatment options for SCLC did not change significantly during the period tumor samples were collected; therefore, the tissue collection period is not likely to be a confounder for association between biomarker expression status and OS. Notably, this assumption was confirmed by univariate analysis from Cox proportional hazard modeling.

In conclusion, approximately one-third of patients with ES-SCLC in Denmark were identified as being positive for both PD-L1 and PD-L2 using CPS ≥1 as the threshold for positivity. Expression of PD-L1 and PD-L2 in tumor samples was found to be highly congruent, and patients with PD-L1−positive and PD-L2−positive tumors were found to have longer OS than those with PD-L1−negative and PD-L2−negative tumors. PD-L2 does not provide additional prognostic value independent of PD-L1.

## Data availability statement

Merck Sharp & Dohme LLC., a subsidiary of Merck & Co., Inc., Rahway, NJ, USA (MSD) is committed to providing qualified scientific researchers access to anonymized data and clinical study reports from the company’s clinical trials for the purpose of conducting legitimate scientific research. MSD is also obligated to protect the rights and privacy of trial participants and, as such, has a procedure in place for evaluating and fulfilling requests for sharing company clinical trial data with qualified external scientific researchers. The MSD data sharing website (available at: http://engagezone.msd.com/ds_documentation.php) outlines the process and requirements for submitting a data request. Applications will be promptly assessed for completeness and policy compliance. Feasible requests will be reviewed by a committee of MSD subject matter experts to assess the scientific validity of the request and the qualifications of the requestors. In line with data privacy legislation, submitters of approved requests must enter into a standard data-sharing agreement with MSD before data access is granted. Data will be made available for request after product approval in the US and EU or after product development is discontinued. There are circumstances that may prevent MSD from sharing requested data, including country or region-specific regulations. If the request is declined, it will be communicated to the investigator. Access to genetic or exploratory biomarker data requires a detailed, hypothesis-driven statistical analysis plan that is collaboratively developed by the requestor and MSD subject matter experts; after approval of the statistical analysis plan and execution of a data-sharing agreement, MSD will either perform the proposed analyses and share the results with the requestor or will construct biomarker covariates and add them to a file with clinical data that is uploaded to an analysis portal so that the requestor can perform the proposed analyses.

## Ethics statement

The studies involving humans were approved by Regional Ethics Committee Videnskabsetiske Komitéer for Region Midtjylland. The studies were conducted in accordance with the local legislation and institutional requirements. The participants provided their written informed consent to participate in this study.

## Author contributions

TS, JBG, PM, ACD, MA, and MCP contributed to conceptualization. TS, PM, ACD, MA, and KZ contributed to data curation. TS, JBG, and MA contributed to methodology. TS and PM contributed to investigation. TS, JBG, and PM contributed to resources. KZ contributed to project administration. TS, JBG, PM, ACD, MA, MCP, and KZ contributed to writing-reviewing and editing. All authors contributed to the article and approved the submitted version.
