# Supplementary material for: Molecular epidemiology study of programmed death ligand 1 and ligand 2 protein expression assessed by immunohistochemistry in extensive-stage small-cell lung cancer
Source: Front Oncol. 2024 Jan 9;13:1225820. doi: 10.3389/fonc.2023.1225820 (PMC10807038; doi:10.3389/fonc.2023.1225820)

Supplementary Material

Table S1. Demographic and Clinicopathologic Characteristics by PD-L1 Protein Expression Status Among Patients With ES-SCLC in Denmark

		PD-L1 Expression	
	**All patients[a]** **N = 80**	**CPS ≥1[a]** **N = 25**	**CPS<1[a]** **N = 55**
Age at SCLC diagnosis, y			
<65	39 (49)	13 (52)	26 (47)
≥65	41 (51)	12 (48)	29 (53)
Age at ES-SCLC diagnosis, y			
<65	38 (48)	13 (52)	25 (46)
≥65	42 (53)	12 (48)	30 (55)
Male	40 (50)	13 (52)	27 (49)
Tissue collection date			
2000–2009	23 (29)	5 (20)	18 (33)
2010–2014	57 (71)	20 (80)	37 (67)
Smoking history			
Current	38 (48)	11 (44)	27 (49)
Former	18 (23)	7 (28)	11 (20)
Unknown	24 (30)	7 (28)	17 (31)
ECOG PS			
0	10 (13)	4 (16)	6 (11)

1	22 (28)	8 (32)	14 (26)
≥2	27 (34)	5 (20)	22 (40)
Unknown	21 (26)	8 (32)	13 (24)
General stage of SCLC at diagnosis			
Limited (I, II, III)	19 (24)	10 (40)	9 (16)
Extensive (IV)	61 (76)	15 (60)	46 (84)
Stage of SCLC at time of specimen collection			
LS-SCLC	10 (13)	5 (20)	5 (9)
LS-SCLC with progression to ES	9 (11)	5 (20)	4 (7)
ES-SCLC at initial diagnosis	61 (76)	15 (60)	46 (84)
Liver metastasis			
Yes	18 (23)	4 (16)	14 (26)
No	62 (78)	21 (84)	41 (75)
Brain metastasis			
Yes	21 (26)	7 (28)	14 (26)
No	59 (74)	18 (72)	41 (75)
Bone metastasis			
Yes	21 (26)	4 (16)	17 (31)
No	59 (74)	21 (84)	38 (69)
Pleura metastasis			
Yes	2 (3)	0	2 (4)

No	78 (98)	25 (100)	53 (96)
Peritoneum metastasis			
Yes	2 (3)	0	2 (4)
No	78 (98)	25 (100)	53 (96)
Skin metastasis			
Yes	3 (4)	2 (8)	1 (2)
No	77 (96)	23 (92)	54 (98)
Distant lymph node metastasis			
Yes	27 (34)	11 (44)	16 (29)
No	53 (66)	14 (56)	39 (71)
Other metastasis			
Yes	19 (24)	7 (28)	12 (22)
No	61 (76)	18 (72)	43 (78)
Any metastases			
Yes	76 (95)	24 (96)	52 (95)
No	4 (5)	1 (4)	3 (6)
Any liver or brain metastases			
Yes	37 (46)	9 (36)	28 (51)
No	43 (54)	16 (64)	27 (49)
Treatment prior to specimen collection			
Chemotherapy exposed	9 (11)	4 (16)	5 (9)

Chemotherapy naive	69 (86)	21 (84)	48 (87)
Unknown	2 (3)	0	2 (4)
Prior or current chemotherapy			
Yes	57 (71)	20 (80)	37 (67)
No	10 (13)	4 (16)	6 (11)
Unknown	13 (16)	1 (4)	12 (22)
Etoposide-platinum−containing regimen			
Subjects with data, n	57	20	37
Yes	53 (93)	20 (100)	33 (89)
No	4 (7)	0	4 (11)
Irinotecan-containing regimen			
Subjects with data, n	57	20	37
No	57 (100)	20 (100)	37 (100)
1L chemotherapy			
Yes	57 (71)	20 (80)	37 (67)
No	20 (25)	5 (20)	15 (27)
Unknown	3 (4)	0	3 (6)
Response to 1L chemotherapy			
Subjects with data, n	57	20	37
Sensitive	40 (70)	17 (85)	23 (62)
Insensitive	7 (12)	2 (10)	5 (14)
Unknown	10 (18)	1 (5)	9 (24)

Previous radiation therapy for SCLC			
Yes	5 (6)	2 (8)	3 (6)
No	62 (78)	22 (88)	40 (73)
Unknown	13 (16)	1 (4)	12 (22)
Lactate dehydrogenase			
Normal	18 (23)	9 (36)	9 (16)
Elevated	27 (34)	4 (16)	23 (42)
Unknown	35 (44)	12 (48)	23 (42)

1L, first line; ECOG PS, Eastern Cooperative Oncology Group performance score; ES, extensive stage; LS, limited stage; PD-L1, programmed death ligand 1; SCLC, small-cell lung cancer.
[a]All data are n (%) unless otherwise noted.

Table S2. Demographic and Clinicopathologic Characteristics by PD-L2 Protein Expression Status Among Patients With ES-SCLC in Denmark

		PD-L2 Expression	
	**All Patients[a]<br>N = 80**	**CPS ≥1[a]<br>N = 29**	**CPS <1[a]<br>N = 51**
Age at SCLC diagnosis, y			
<65	39 (49)	14 (48)	25 (49)
≥65	41 (51)	15 (52)	26 (51)
Age at ES-SCLC diagnosis, y			
<65	38 (48)	14 (48)	24 (47)
≥65	42 (53)	15 (52)	27 (53)
Male	40 (50)	16 (55)	24 (47)
Tissue collection date			
2000–2009	23 (29)	7 (24)	16 (31)
2010–2014	57 (71)	22 (76)	35 (69)
Smoking history			
Current	38 (48)	14 (48)	24 (47)
Former	18 (23)	10 (35)	8 (16)
Unknown	24 (30)	5 (17)	19 (37)
ECOG PS			
0	10 (13)	4 (14)	6 (12)
1	22 (28)	11 (38)	11 (22)

≥2	27 (34)	8 (28)	19 (37)
Unknown	21 (26)	6 (21)	15 (29)
General stage of SCLC at diagnosis			
Limited (I, II, III)	19 (24)	8 (28)	11 (22)
Extensive (IV)	61 (76)	21 (72)	40 (78)
Stage of SCLC at time of specimen collection			
LS-SCLC	10 (13)	3 (10)	7 (14)
LS-SCLC with progression to ES	9 (11)	5 (17)	4 (8)
ES-SCLC at initial diagnosis	61 (76)	21 (72)	40 (78)
Liver metastasis			
Yes	18 (23)	5 (17)	13 (26)
No	62 (78)	24 (83)	38 (75)
Brain metastasis			
Yes	21 (26)	9 (31)	12 (24)
No	59 (74)	20 (69)	39 (77)
Bone metastasis			
Yes	21 (26)	7 (24)	14 (28)
No	59 (74)	22 (76)	37 (73)
Pleura metastasis			
Yes	2 (3)	0	2 (4)
No	78 (98)	29 (100)	49 (96)

Peritoneum metastasis			
Yes	2 (3)	0	2 (4)
No	78 (98)	29 (100)	49 (96)
Skin metastasis			
Yes	3 (4)	1 (3)	2 (4)
No	77 (96)	28 (97)	49 (96)
Distant lymph node metastasis			
Yes	27 (34)	14 (48)	13 (26)
No	53 (66)	15 (52)	38 (75)
Other metastasis			
Yes	19 (24)	6 (21)	13 (26)
No	61 (76)	23 (79)	38 (75)
Any metastases			
Yes	76 (95)	28 (97)	48 (94)
No	4 (5)	1 (3)	3 (6)
Any liver or brain metastases			
Yes	37 (46)	12 (41)	25 (49)
No	43 (54)	17 (59)	26 (51)
Treatment prior to specimen collection			
Chemotherapy exposed	9 (11)	4 (14)	5 (10)
Chemotherapy naive	69 (86)	25 (86)	44 (86)

Unknown	2 (3)	0 (0.0)	2 (4)
Prior or current chemotherapy			
Yes	57 (71)	25 (86)	32 (63)
No	10 (13)	3 (10)	7 (14)
Unknown	13 (16)	1 (3)	12 (24)
Etoposide-platinum–containing regimen			
Subjects with data, n	57	25	32
Yes	53 (93)	25 (100)	28 (88)
No	4 (7)	0	4 (13)
Irinotecan-containing regimen			
Subjects with data, n	57	25	32
No	57 (100)	25 (100)	32 (100)
1L chemotherapy			
Yes	57 (71)	25 (86)	32 (63)
No	20 (25)	4 (14)	16 (31)
Unknown	3 (4)	0	3 (6)
Response to 1L chemotherapy			
Subjects with data, n	57	25	32
Sensitive	40 (70)	21 (84)	19 (59)
Insensitive	7 (12)	2 (8)	5 (16)
Unknown	10 (18)	2 (8)	8 (25)

Previous radiation therapy for SCLC			
Yes	5 (6)	3 (10)	2 (4)
No	62 (78)	25 (86)	37 (73)
Unknown	13 (16)	1 (3)	12 (24)
Lactate dehydrogenase			
Normal	18 (23)	9 (31)	9 (18)
Elevated	27 (34)	5 (17)	22 (43)
Unknown	35 (44)	15 (52)	20 (39)

1L, first line; ECOG PS, Eastern Cooperative Oncology Group performance score; ES, extensive stage; LS, limited stage; PD-L2, programmed death ligand 2; SCLC, small-cell lung cancer.
[a]All data are n (%) unless otherwise noted.

Table S3. Demographic and Clinicopathologic Characteristics by PD-L1/PD-L2 Coexpression Status Among Patients With ES-SCLC in Denmark

		PD-L1 and PD-L2 Coexpression	
	All Patients[a] N = 68	PD-L1 and PD-L2 With CPS ≥1 (Double Positive)[a] N = 21	PD-L1 and PD-L2 With CPS <1 (Double Negative)[a] N = 47
Age at SCLC diagnosis, y			
<65	32 (47)	10 (48)	22 (47)
≥65	36 (53)	11 (52)	25 (53)
Age at ES-SCLC diagnosis, y			
<65	31 (46)	10 (48)	21 (45)
≥65	37 (54)	11 (52)	26 (55)
Male	31 (46)	10 (48)	21 (45)
Tissue collection date			
2000–2009	21 (31)	5 (24)	16 (34)
2010–2014	47 (69)	16 (76)	31 (66)
Smoking history			
Current	35 (52)	11 (52)	24 (51)
Former	13 (19)	6 (29)	7 (15)
Unknown	20 (29)	4 (19)	16 (34)
ECOG PS			
0	10 (15)	4 (19)	6 (13)

1	17 (25)	7 (33)	10 (21)
≥2	24 (35)	5 (24)	19 (40)
Unknown	17 (25)	5 (24)	12 (26)
General stage of SCLC at diagnosis			
Limited (I, II, III)	15 (22)	7 (33)	8 (17)
Extensive (IV)	53 (78)	14 (67)	39 (83)
Stage of SCLC at time of specimen collection			
LS-SCLC	8 (12)	3 (14)	5 (11)
LS-SCLC with progression to ES	7 (10)	4 (19)	3 (6)
ES-SCLC at initial diagnosis	53 (78)	14 (67)	39 (83)
Liver metastasis			
Yes	17 (25)	4 (19)	13 (28)
No	51 (75)	17 (81)	34 (72)
Brain metastasis			
Yes	17 (25)	6 (29)	11 (23)
No	51 (75)	15 (71)	36 (77)
Bone metastasis			
Yes	18 (27)	4 (19)	14 (30)
No	50 (74)	17 (81)	33 (70)
Pleura metastasis			
Yes	2 (3)	0	2 (4)

No	66 (97)	21 (100)	45 (96)
Peritoneum metastasis			
Yes	2 (3)	0	2 (4)
No	66 (97)	21 (100)	45 (96)
Skin metastasis			
Yes	2 (3)	1 (5)	1 (2)
No	66 (97)	20 (95)	46 (98)
Distant lymph node metastasis			
Yes	22 (32)	10 (48)	12 (26)
No	46 (68)	11 (52)	35 (75)
Other metastasis			
Yes	16 (24)	5 (24)	11 (23)
No	52 (77)	16 (76)	36 (77)
Any metastases			
Yes	64 (94)	20 (95)	44 (94)
No	4 (6)	1 (5)	3 (6)
Any liver or brain metastases			
Yes	32 (47)	8 (38)	24 (51)
No	36 (53)	13 (62)	23 (49)
Treatment prior to specimen collection			
Chemotherapy exposed	7 (10)	3 (14)	4 (9)

Chemotherapy naive	59 (87)	18 (86)	41 (87)
Unknown	2 (3)	0	2 (4)
Prior or current chemotherapy			
Yes	46 (68)	17 (81)	29 (62)
No	9 (13)	3 (14)	6 (13)
Unknown	13 (19)	1 (5)	12 (26)
Etoposide-platinum−containing regimen			
Subjects with data, n	46	17	29
Yes	42 (91)	17 (100)	25 (86)
No	4 (9)	0	4 (14)
Irinotecan-containing regimen			
Subjects with data, n	46	17	29
No	46 (100)	17 (100)	29 (100)
1L chemotherapy			
Yes	46 (68)	17 (81)	29 (62)
No	19 (28)	4 (19)	15 (32)
Unknown	3 (4)	0	3 (6)
Response to 1L chemotherapy			
Subjects with data, n	46	17	29
Sensitive	32 (70)	15 (88)	17 (59)
Insensitive	5 (11)	1 (6)	4 (14)
Unknown	9 (20)	1 (6)	8 (28)

Previous radiation therapy for SCLC			
Yes	4 (6)	2 (10)	2 (4)
No	51 (75)	18 (86)	33 (70)
Unknown	13 (19)	1 (5)	12 (26)
Lactate dehydrogenase			
Normal	14 (21)	7 (33)	7 (15)
Elevated	24 (35)	3 (14)	21 (45)
Unknown	30 (44)	11 (52)	19 (40)

1L, first line; ECOG PS, Eastern Cooperative Oncology Group performance score; ES, extensive stage; LS, limited stage; PD-L1, programmed death ligand 1; PD-L2, programmed death ligand 2; SCLC, small-cell lung cancer.

[a]All data are n (%) unless otherwise noted.

Figure S1. IHC staining of the entire slide (x20) for PD-L1 and PD-L2 in SCLC tumor tissue samples. A) PD-L1 negative (CPS = 0), B) PD-L2 negative (CPS = 0), C) PD-L1 positive (CPS = 40), and D) PD-L2 positive (CPS = 40). Arrows indicate representative areas of positive staining for PD-L1 (panel C) and PD-L2 (panel D).

A)

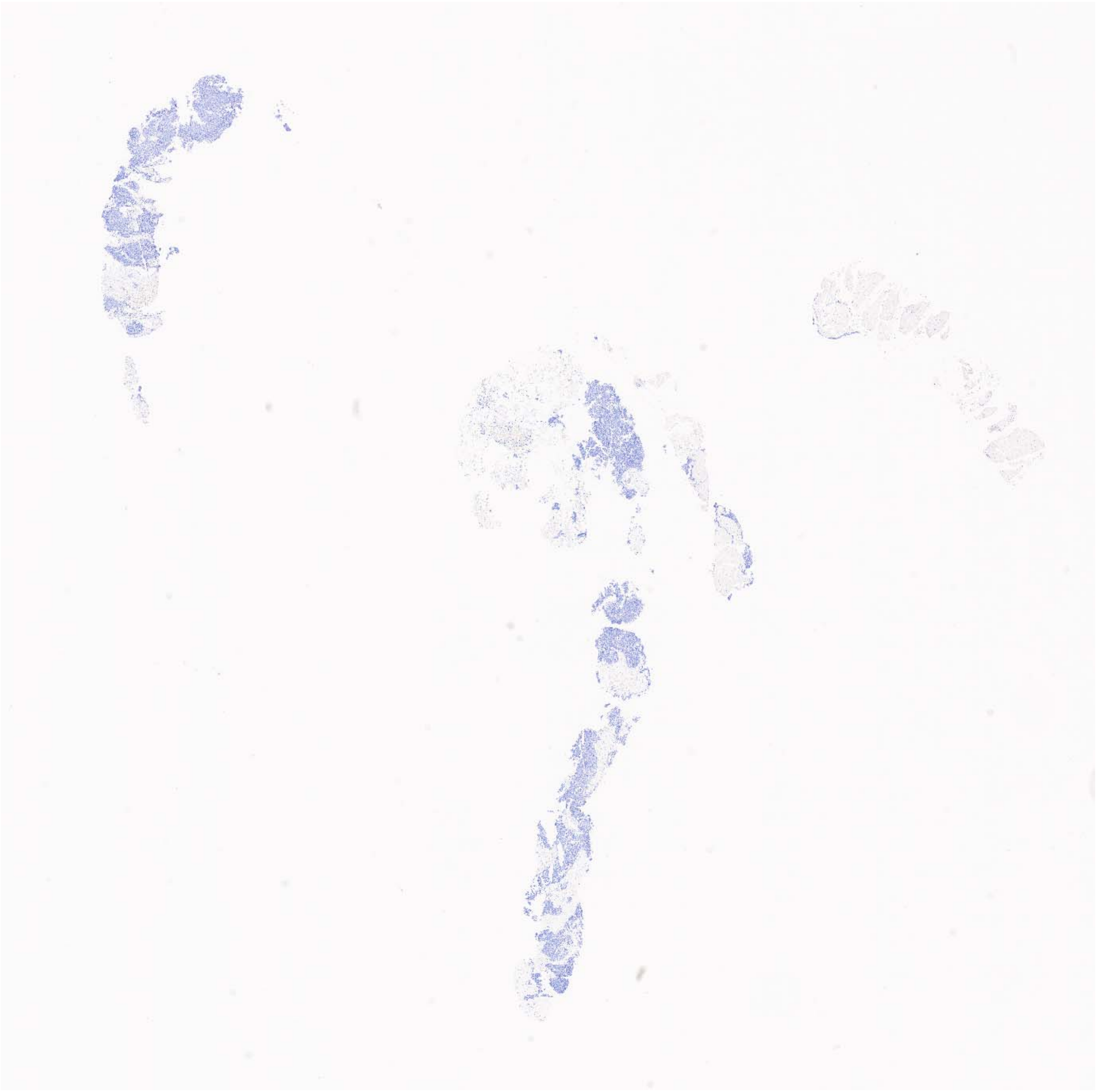

B)

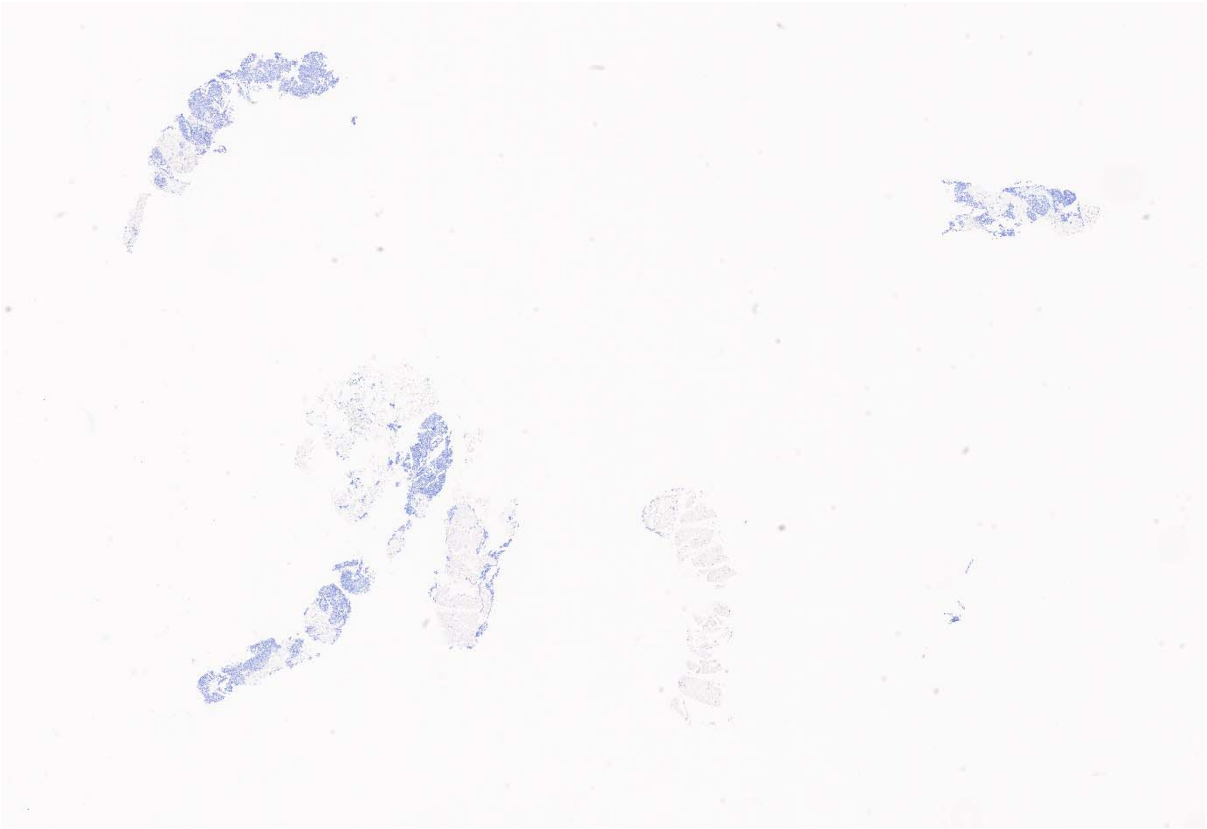

C)

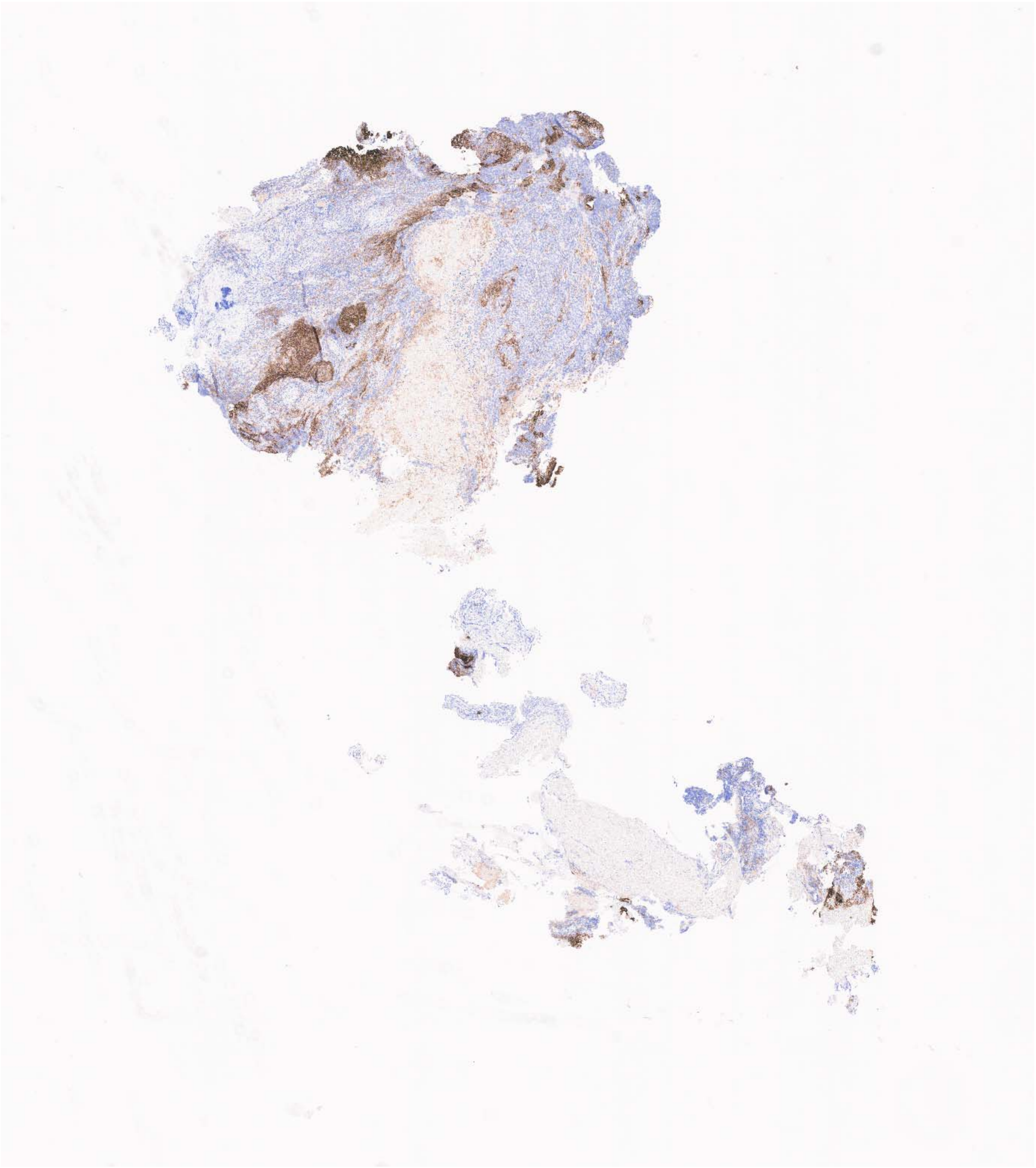

D)

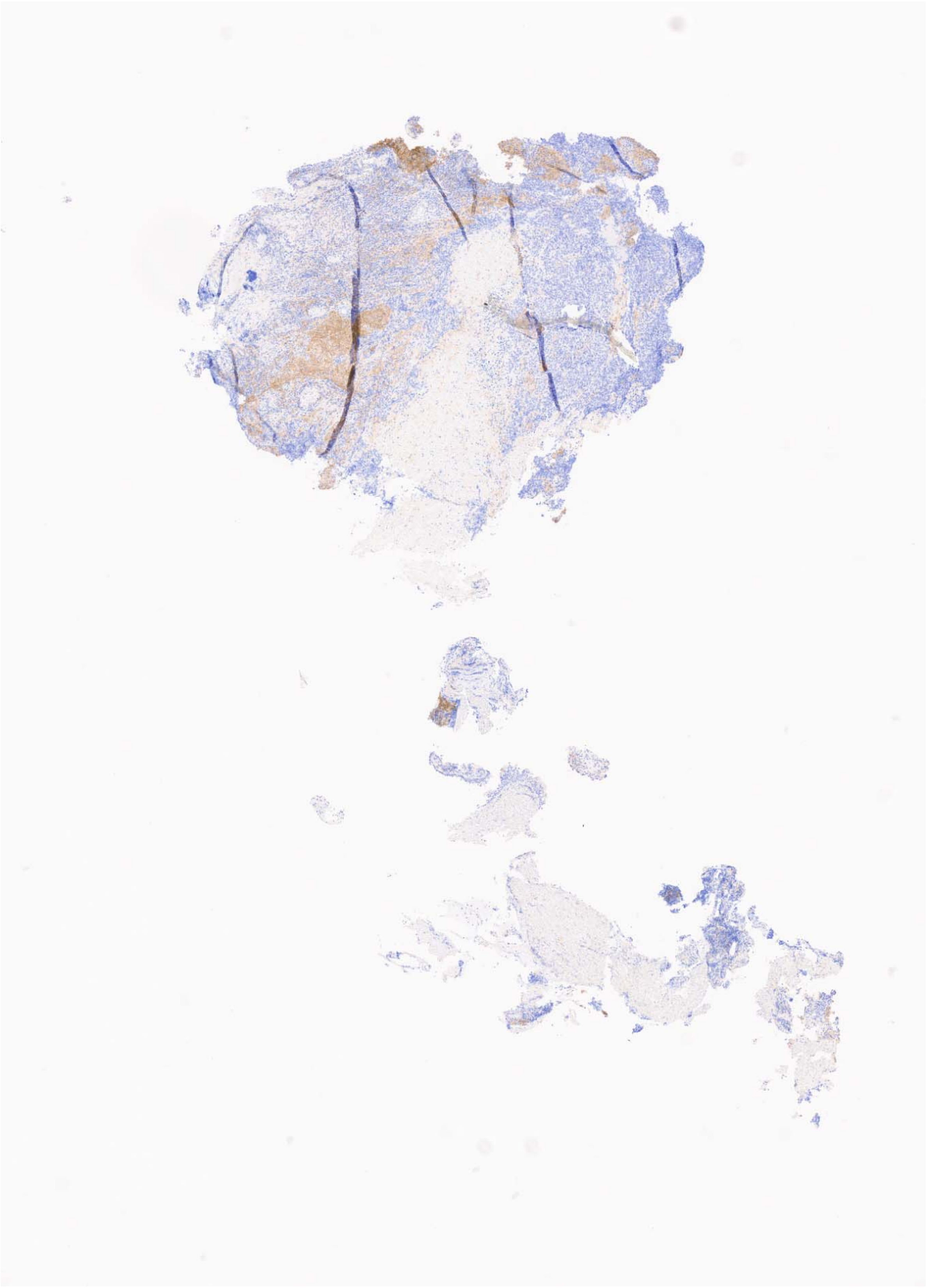

Figure S2. Kaplan-Meier estimates of overall survival by PD-L1 expression status in all patients with ES-SCLC in Denmark. CPS, combined positive score; ES-SCLC, extensive-stage small-cell lung cancer; PD-L1, programmed death ligand 1.

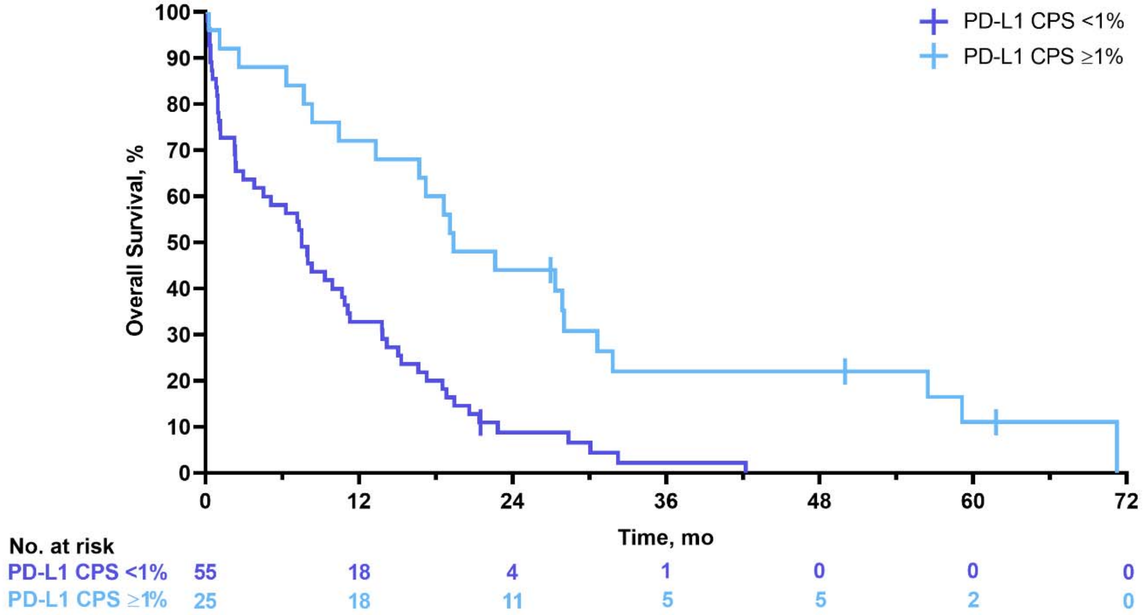

Figure S3. Kaplan-Meier estimates of overall survival by PD-L2 expression status in all patients with ES-SCLC in Denmark. CPS, combined positive score; ES-SCLC, extensive-stage small-cell lung cancer; PD-L2, programmed death ligand 2.

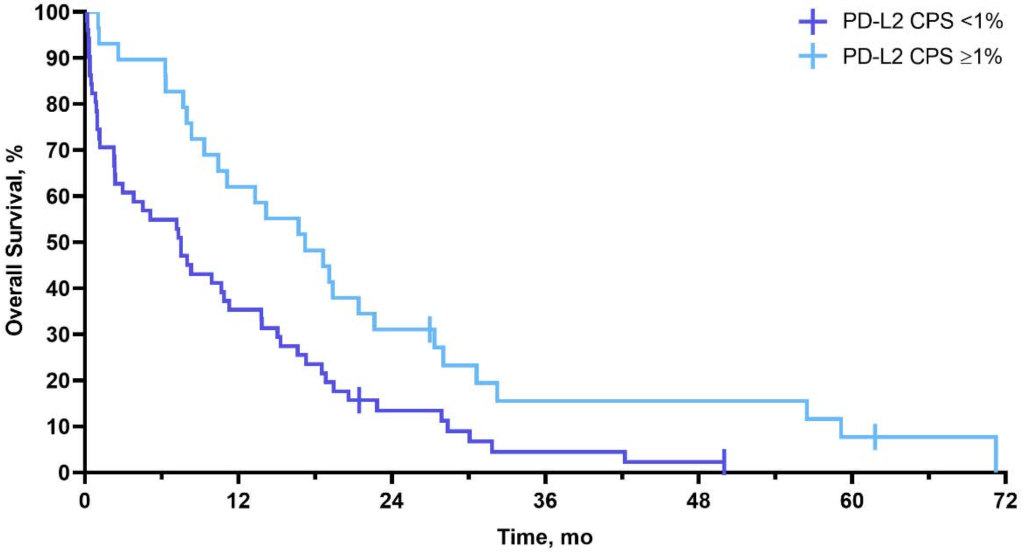

No. at risk							
PD-L2 CPS <1%	51	18	6	2	1	0	0
PD-L2 CPS ≥1%	29	18	9	4	4	2	0

Figure S4. Kaplan-Meier estimates of overall survival by PD-L1 and PD-L2 coexpression status in all patients with ES-SCLC in Denmark. CPS, combined positive score; ES-SCLC, extensive-stage small-cell lung cancer; PD-L1, programmed death ligand 1; PD-L2, programmed death ligand 2.

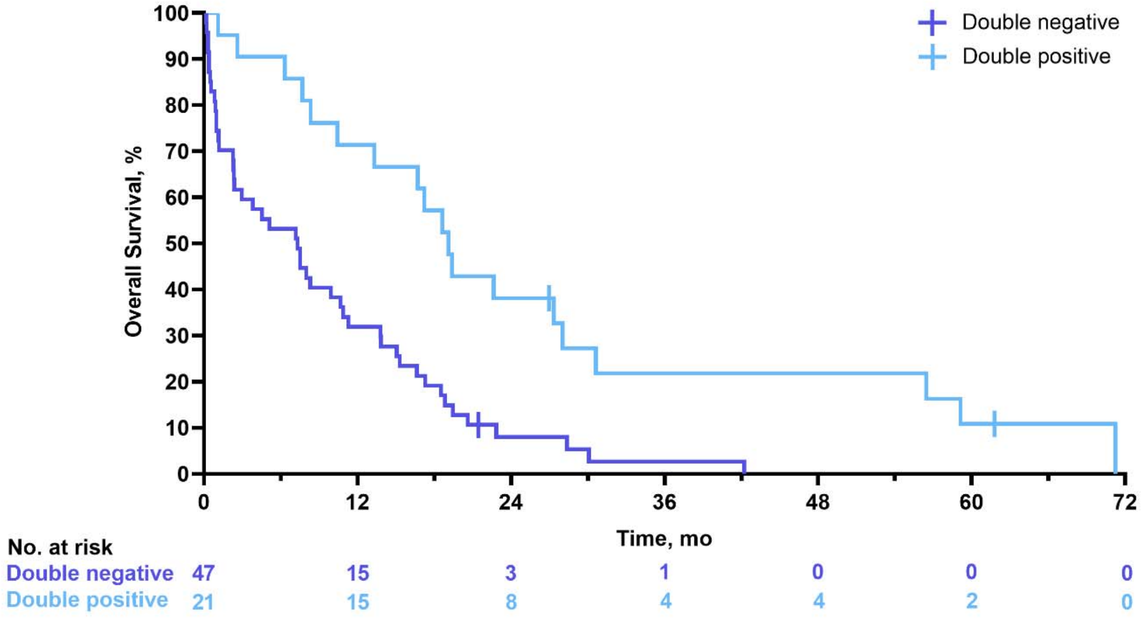

Supplement: Supplementary file 1 [file DataSheet_1.pdf]
